# Whole mitochondrial genome sequencing of domestic horses reveals incorporation of extensive wild horse diversity during domestication

**DOI:** 10.1186/1471-2148-11-328

**Published:** 2011-11-14

**Authors:** Sebastian Lippold, Nicholas J Matzke, Monika Reissmann, Michael Hofreiter

**Affiliations:** 1 Department of Evolutionary Genetics, Max Planck Institute for Evolutionary Anthropology, Deutscher Platz 6, 04103 Leipzig, Germany; 2 Center for Theoretical Evolutionary Genomics, Department of Integrative Biology, University of California, Berkeley, 4151 Valley Life Sciences Building, Berkeley, CA, USA; 3 Department for Crop and Animal Sciences, Humboldt University Berlin, Invalidenstr. 42, 10115 Berlin, Germany; 4 Department of Biology, University of York, Wentworth Way, Heslington, York YO10 5DD, UK

## Abstract

**Background:**

DNA target enrichment by micro-array capture combined with high throughput sequencing technologies provides the possibility to obtain large amounts of sequence data (e.g. whole mitochondrial DNA genomes) from multiple individuals at relatively low costs. Previously, whole mitochondrial genome data for domestic horses (*Equus caballus*) were limited to only a few specimens and only short parts of the mtDNA genome (especially the hypervariable region) were investigated for larger sample sets.

**Results:**

In this study we investigated whole mitochondrial genomes of 59 domestic horses from 44 breeds and a single Przewalski horse (*Equus przewalski*) using a recently described multiplex micro-array capture approach. We found 473 variable positions within the domestic horses, 292 of which are parsimony-informative, providing a well resolved phylogenetic tree. Our divergence time estimate suggests that the mitochondrial genomes of modern horse breeds shared a common ancestor around 93,000 years ago and no later than 38,000 years ago. A Bayesian skyline plot (BSP) reveals a significant population expansion beginning 6,000-8,000 years ago with an ongoing exponential growth until the present, similar to other domestic animal species. Our data further suggest that a large sample of wild horse diversity was incorporated into the domestic population; specifically, at least 46 of the mtDNA lineages observed in domestic horses (73%) already existed before the beginning of domestication about 5,000 years ago.

**Conclusions:**

Our study provides a window into the maternal origins of extant domestic horses and confirms that modern domestic breeds present a wide sample of the mtDNA diversity found in ancestral, now extinct, wild horse populations. The data obtained allow us to detect a population expansion event coinciding with the beginning of domestication and to estimate both the minimum number of female horses incorporated into the domestic gene pool and the time depth of the domestic horse mtDNA gene pool.

## Background

Among domesticated species, the horse represents the last one of major importance to become domesticated. The domestication of the horse had a lasting impact on human societies, by increasing mobility and trade, influencing human lifestyles and profoundly changing warfare. In turn, artificial selection by humans shaped the genetic diversity in horse populations, resulting in the variation observed in modern horse phenotypes and breeds. Several studies have investigated the genetic relationship among horse breeds using mitochondrial sequences as a marker [1-13] (for a review see [14]). Most of the studies published so far used short, but highly variable fragments from the mitochondrial hypervariable region (HVR). Based on a 616 base pair (bp) fragment of the mtDNA control region from 37 domestic horses, Vilà *et al *[9] suggested the existence of at least six divergent sequence clades. In another study on a 247 bp fragment of the hypervariable region from a worldwide sample of 652 horses, up to 17 major haplogroups were identified in a phylogenetic network [8]. In the latter study, several of these haplogroups were associated with breeds and/or geographical areas (e.g. C1 for northern European ponies). A number of additional studies extended the picture of horse mtDNA diversity and distribution by including additional breeds from Asia (especially from China and Mongolia) [1-3] and it was proposed that there is evidence of a weak phylogeographic pattern [4].

However, it is notable that all previous mitochondrial studies had little to no statistical support for their inferred phylogenetic trees, with most of the nodes remaining unresolved. The short sequence length and small evolutionary distance between horse breeds results in a low number of phylogenetically informative sites, and this has so far prevented high statistical support for most of the nodes within the mtDNA phylogenetic tree of the domestic horse. The introduction and continued development of next generation sequencing (NGS) now allows the acquisition of much larger sequence data sets in shorter time and at lower costs compared to what was possible using classical Sanger sequencing. For mitochondrial DNA, sequencing the complete mitochondrial genome has been shown to improve phylogenetic resolution for the marker both between and within species [15-20]. The phylogenetic relationships of different groups of cave bears [16] as well as of killer whales [15] were in fact only resolved by sequencing complete mtDNA genomes.

While these studies used PCR to enrich for mtDNA sequencing, we have used a novel approach in which barcoded sequencing libraries from multiple samples are pooled [21] and the mtDNA genomes are enriched by hybridization capture on a micro array [22] and sequenced on an Illumina (Solexa) GAII sequencing machine [21].

## Results

### Sequence data analysis and alignment

We sequenced 59 horse samples from 44 breeds and a Przewalski horse (Additional file 1, Table S1) using an Illumina/Solexa GA II system after enriching for complete mitochondrial genomes by multiplex micro-array capture. On average, 17,474 sequence reads per sample mapped to the mtDNA genome (Additional file 1, Table S2). The minimum number of reads per sample was 5,666 (for Vjatka horse), while the maximum number was 30,368 (for Kustanai horse). The average sequence coverage per position after duplicate removal was on average 53-fold coverage and ranged from 14-fold for Vjatka horse to 82-fold for Clydesdale, respectively (Additional file 1, Table S2).

A consensus sequence for each of the samples was called based on the criteria described in the methods section. Positions not fulfilling these criteria were called as 'N' (Additional file 1, Table S2). The maximum number of missing positions was observed for the English Thoroughbred (124 positions), which are 0.75% of the investigated mtDNA-genome positions. All consensus sequences were submitted to NCBI GenBank (accession numbers see Additional file 1, Table S1). The 60 consensus sequences, six modern horse sequences from GenBank and a full mtDNA genome sequence for the donkey (Additional file 1, Table S3) were aligned using clustalW [23]. Thirteen positions (1-3, 16,121, 16,127, 16,128, 16,364, 16,371, 16,656-16,660) that show a missing base call in at least three samples were removed from the alignment.

Preliminary phylogenetic analysis showed that three of the GenBank-derived sequences ("jeju", "debao", and "zhongdian", respectively accession numbers [GenBank: AY584828.1], [GenBank: EU939445], and [GenBank: EF597512.1]) exhibited unusually long branches, and strong departure from the clocklike evolution of the rest of sequences (Additional file 1, Figure S1). The same pattern was observed on several different MrBayes runs, as well as maximum likelihood runs with PHYML and RAxML. This behavior might indicate contamination of these sequences by nuclear copies of the mtDNA (numts), or some other problem with these sequences. Therefore, they were eliminated from the alignment and excluded from the remainder of the analysis.

The final alignment used for phylogenetic analysis consisted of 64 sequences and 16,414 nucleotide positions, of which 473 were variable and 292 were parsimony-informative within horses. Each of the sequences represents a unique haplotype.

### Phylogenetic analysis

**Figure 1:**
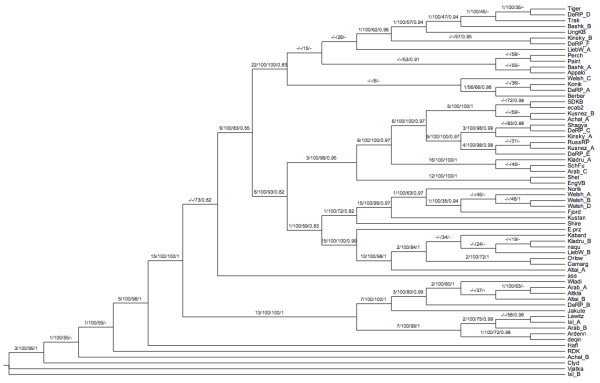
**Cladogram of the fully resolved PHYML tree estimated using the Modeltest-specified HKY+I+G substitution model, with support values from each major type of analysis displayed on each bipartition**. Each bipartition shows the Bremer support, bootstrap support on the MP strict consensus tree, bootstrap support on the PHYML tree, and Bayesian posterior credibility from the MrBayes GTR+I+G analysis. A dash is shown if Bremer support is 0 or negative, if MP bootstraps are below 50%, if there was no bootstrap support for the bipartition on the PHYML tree, or if the Bayesian posterior credibility was below 0.5 for the bipartition in question.

Summaries of maximum parsimony (MP), maximum likelihood (ML) and Bayesian phylogenetic analyses are available in Additional file 1, Table S4. In general, good resolution was achieved, with many nodes resolved with high bootstrap and Bremer support even with strict consensus trees; however, some of the very closely related mtDNA lineages were not resolved (Figure 1).

In most of the cases when two or more individuals were sequenced from the same breed (Akhal-Teke, Altai, Arab, Bashkir Curly, Icelandic horse, Kinsky horse, Kladruber, Kuznet, Liebenthaler, Rhineland Heavy Draft and Welsh Pony), their mtDNA sequences fall on widely-spaced tips of the tree. The only exception to this observation consists of three Welsh Pony sequences which form a clade, although even here a fourth Welsh Pony sequence (Welsh_C Section B) falls outside this clade. These results represent strong evidence that many breeds do not have a single maternal origin and that they retain much of the ancestral mtDNA variation originally found in the wild, pre-domestication populations spread across Eurasia [8,9].

### Divergence times

Tests of the hypothesis of a strict, global molecular clock using likelihood ratio tests (Additional file 1, Table S5) either rejected the clock at p < 0.05 but at barely significant p-values (PAUP clock analyses, p-values ranging from 0.021 to 0.049), or failed to reject the clock (PAML based ML analysis, p = 0.057). As tests of a strict clock often reject the hypothesis even when there is clocklike behavior and as, given the low sequence divergence, the young age of the horse clade, and the closely-clocklike appearance of phylogenetic trees displaying un-calibrated molecular branch-lengths, clocklike behavior is likely in this situation, the decision was made to accept the molecular clock hypothesis for the purposes of further analysis.

**Figure 2:**
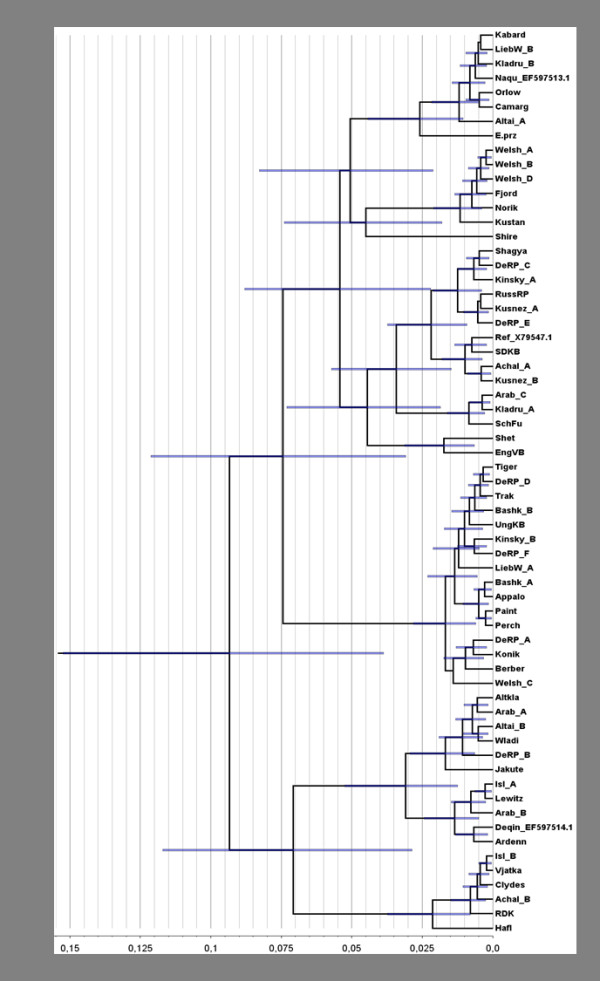
**95% credibility intervals on node ages as estimated by BEAST using a normally-distributed prior on the horse/ass divergence time centered at 2.25 mybp with the standard deviation set to 0.3125 my**.

R8s analysis using the Langley-Fitch method (strict clock) yielded a maximum divergence time for the mtDNAs of the horse breeds of 160,000 years and minimum time of 50,000 years. BEAST, using a normally-distributed prior on the horse/ass divergence time, estimated 93,400 with a 95% credibility interval of 152,000-38,800 years and a substitution rate of 7.39^-02 ^substitutions/site/Mya (95% HPD: 2.49^-02^-1.60^-01^). The tree with the mean ages (nodes) and the 95% credibility intervals (blue bars) is shown in Figure 2.

### Demographic history

**Figure 3:**
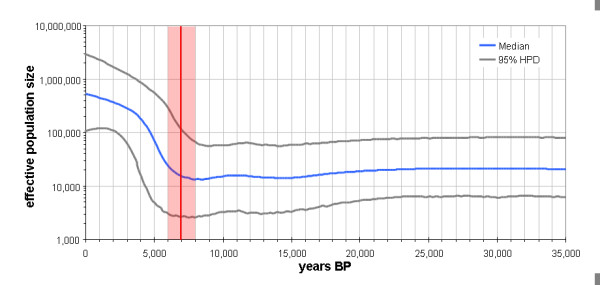
**Bayesian Skyline Plot of effective population size through time based on the whole mtDNA sequence from 63 horses**. The beginning of the recent effective population size expansion is marked in red (median 7,000 years BP).

In order to investigate changes in maternal population size through time a Bayesian analysis using the Bayesian Skyline model [24] was carried out. The Bayes factor (BF) computed via importance sampling [25] with Tracer [26] favoured the BSP model over the constant size model (log10 BF = 1.496; [27]). The BSP (Figure 3) indicates a constant population size until ~ 7,000 years BP (95% HPD 6,000-8,000 years BP) followed by a continued population expansion until the present and a current maternal effective population size of ~530,000.

## Discussion

In many domesticated animal species, the analysis of mtDNA has for a long time suffered from limited phylogenetic resolution offered by the short mtDNA fragments used. This issue is particularly severe in horses, which display one of the highest amounts of mtDNA diversity for any domesticated animal. Recently, the analysis of complete mitochondrial genomes in dogs [28] and cattle [18,29] has revived the use of mtDNA in studying domestication. These studies showed that the use of complete mtDNA genomes improved not only phylogenetic resolution but also resulted in more precise dates for the divergence of the different maternal lineages, and have improved our understanding of the geographical origins of both dogs and cattle, although the geographical origin of dogs inferred from the mitochondrial data has been challenged based on analyses of autosomal nuclear DNA data [30].

An increased phylogenetic resolution could therefore potentially also result in a better phylogeographical or breed specific resolution of horse mtDNA sequences. However, this is not the case. Although we have only a small number of breeds with at least two sampled individuals and any conclusion about the intra-breed variation based on our data is therefore inherently limited, their respective sequences are generally spread across the tree with no evidence that mtDNA sequences from the same breed are more closely related than what would be expected by chance. This indicates that mitochondrial DNA alone is unlikely to resolve the geographical origin of horse domestication. Given the relatively recent origin of modern horse breeds and the extensive trade of horses as well as their use as a means of long-distance transport, this result is, however, not entirely surprising. Resolving the timing and geographical origin of horse domestication will therefore require the use of alternative genetic markers such as autosomal single-nucleotide polymorphism provided by the horse genome sequencing project [31] or phenotypic markers such as coat colour polymorphisms [32] in conjunction with further archaeological studies.

The investigation of the population size through time using the Bayesian Skyline model shows a clear and continued increase in female population size beginning ~7 ky ago, while before this time, population size was constant for tens of thousands of years. Since the climate was relatively stable over the Holocene compared to the glacial period, it seems a reasonable null hypothesis would be that a wild population would be approximately stable during the Holocene. Horse fossil records from the early Holocene are rare in Europe, but become more frequent in the late Atlantic period (7,500-5,750 BP) [33], indicating a population expansion of wild horse population at least in Europe during this period. A similar pattern of a recent expansion in population size was also observed in three domestic bovine species, but not in a single wild bovine species (the African buffalo) investigated for comparison [34]. The similarity in these patterns suggests that the observed steep, recent expansion in horse population size probably indicates the beginning of horse domestication. Since their population size stayed constant for a long time before, despite various climatic fluctuations, the most parsimonious explanation is that domestication was causal for this population increase. This interpretation is supported by the fact that the estimated beginning of expansion (95% HPD 6-8 ky BP) closely coincides with the beginning of horse domestication 5,000 to 5,500 kya as estimated in previous studies [32,35].

Divergence time estimates based on our complete mtDNA sequence data suggest a rather recent ancestor for modern female horse lineages. Both methods used gave similar results with 160,000-50,000 years (r8s) and 152,000-38,000 years (BEAST). These estimates are considerably younger than, and did in fact not overlap at all with, the estimates based on fragments of the mitochondrial d-loop, which range from 630,000-320,000 years [9] to 1,198,000-342,000 years [8], respectively. This result is not entirely surprising as overestimates of the divergence time especially in domestic animals was proposed previously [36]. The fast evolving d-loop is prone to multiple substitutions, especially when the calibration point used for estimates of divergence events relies on a comparatively distantly related species, thereby inflating substitution rate estimates. Including the conserved regions of the mtDNA genome should improve these estimates and therefore give more reliable divergence time estimates.

Although a phylogenetic tree alone does not allow drawing conclusions about the number of mtDNA lineages incorporated into the domestic gene pool, the molecular dates obtained for the divergence events in combination with knowledge about the timing of horse domestication allow some speculations on this issue. If one uses a relatively recent estimate for horse domestication of about 5,000 years ago [32], our data suggest that at least 46 mtDNA lineages and therefore at least as many wild mares, contributed to the domestic gene pool. Given that we only sequenced 60 horses, this is a remarkable high number. If horse domestication took place 11,400 years ago (earliest suggested date in [8]), the number of domesticated mtDNA lineages reduces, but still remains at a comparatively high number of 33, if the point estimate for the divergence dates is used. Jansen *et al*. [8] suggested the incorporation of at least 77 mares into the domestic gene pool. However, their numbers are not directly comparable to our results and should rather be seen as rough estimates. Although the conclusion of Jansen *et al *is based on a much larger sample set, the short length of only 247 bp and the high mutation rate in the d-loop might inflate the calculations. Given that we used 63 horses (including three sequences from NCBI GenBank) in our analyses, our numbers are minimum estimates and screening more modern horses would undoubtedly reveal further domesticated lineages. This is especially true, as several deeply branching lineages in our tree are represented by only a single individual. Therefore it is likely that additional samples would reveal at least some additional mtDNA lineage divergences that predate horse domestication. Thus, when applied to an extensive sampling of horses worldwide this approach promises to yield for the first time an informed estimate about the number of mares that were incorporated into the domestic gene pool.

## Conclusion

Our study provides a window into the maternal origins of extant domestic horses and confirms that modern domestic breeds present a wide sample of the mtDNA diversity found in ancestral, now extinct, wild horse populations. The data obtained allow us to detect a population expansion event coinciding with the beginning of domestication and to estimate both the minimum number of female horses incorporated into the domestic gene pool and the time depth of the domestic horse mtDNA gene pool.

## Methods

### Multiplex array capturing and sequencing

DNA was extracted from hair roots of 60 horses using NucleoSpin Tissue KIT after manufacturer instruction (Macherey-Nagel, Düren, Germany) (Additional file 1, Table S1). Horse samples were taken in correspondence with German animal protection law (Potsdam: 32/44456+11). Genomic DNA (100 μl, conc. 10 ng/μl) was sheared by sonication using a Bioruptor system to a fragment size around 150-250 bp. Next, barcoded Illumina sequencing libraries with a different barcode used for each sample were constructed from the fragmented DNA according to the protocol described in Meyer and Kircher [21]. The 60 barcoded libraries were pooled in equimolar ratio and hybridized on a single 244K custom microarray (Agilent). The microarray was designed that overlapping 60-mer oligonucleotide probes targeting the whole mitochondrial genome were tiled every 15 nucleotides. The repetitive part of the control region (motif GTG CAC CT, pos. 16,129-16,360) was not targeted. Hybridization and sequencing preparations were performed as described in [21,22]. After enrichment, the DNA library was sequenced on the Illumina/Solexa Genome Analyzer II platform (Illumina, San Diego, CA, USA).

### Sequence data analysis

Sequencing runs were analyzed from raw images using the Illumina Genome Analyzer pipeline. Bases were called using Ibis (http://bioinf.eva.mpg.de/Ibis/, [37]) and reads with five or more positions with a PHRED-like quality score below 20 were discarded. Each read was sorted according to the sample specific barcode and the adapter sequence was trimmed. The reads of each sample were aligned against one published mitochondrial genome ([GenBank: X79547.1] [38]) using BWA v0.5.1 (http://bio-bwa.sourceforge.net/, [39]). The BAM alignment files were further processed with SAMtools v0.1.7 (http://samtools.sourceforge.net/, [40]) and alignment statistics including number of mapped reads and average coverage per position were determined. After duplicate removal, for each position in the alignment, the consensus base was called and several quality scores were calculated (i.e. Phred-scaled consensus quality, SNP quality, mapping quality; see http://samtools.sourceforge.net/pileup.shtml) by using the SAMtools "pileup -c" command. The final consensus base was called when the position had a consensus quality score of at least Q30 and a mapping quality score of at least Q20. Further, for base calls that were different to the reference sequence, a SNP quality score of at least Q30 and three-fold coverage in this position was required. Indels were not considered in the base calling process.

From the consensus sequences a multiple sequence alignment was obtained using ClustalW (http://www.ebi.ac.uk/Tools/clustalw2/[23]). We further added all currently available complete mtDNA-genome sequences from horses we found on the NCBI GenBank (http://www.ncbi.nlm.nih.gov/genbank/) and from the wild ass (*Equus asinus*), the closest relative with a fully sequenced mtDNA genome (Additional file 1, Table S3). The repetitive part of the control region (pos. 16,129-16,360 referring to X79547.1 [38]) was masked with "N's" as we also discarded this region in the probe design of the array. Any nucleotide position in the multiple alignment that failed to have information for at least three samples of the alignment (4.47% of the samples) was deleted on the grounds that it was unlikely to represent homology shared across the alignment. A preliminary phylogenetic analysis showed that three of the GenBank-derived sequences ("jeju", "debao", and "zhongdian", respectively accession numbers [GenBank: AY584828.1], [GenBank: EU939445], and [GenBank: EF597512.1]; Additional file 1, Table S3) exhibited unusually long branches, and strong departure from the clocklike evolution of the rest of sequences (Additional file 1, Figure S1). The same pattern was observed on several different MrBayes runs with different parameters, as well as maximum likelihood runs with PHYML and RAxML. This behavior indicates contamination of these sequences by nuclear DNA (numts), or some other problem with these sequences; therefore, they were eliminated from the alignment and excluded from the remainder of the analysis. It might be possible that the long branches of the excluded sequences were due to some "real" effect, such as adaptation to high-altitude environments; however, only one of the three removed sequences, zhongdian, was derived from a study on mitochondrial adaptations in high-altitude Tibetan horse breeds [41], and the other two sequences derived from that study (deqin and naqu), although both from high-altitude locations above 3,000 m in China or Tibet, did not exhibit unusual branch lengths. Therefore, it was judged unlikely that the long branches of the excluded sequences were due to high-altitude adaptation or some similar effect.

The final alignment had 64 sequences and 16,419 nucleotide positions. Initial summary statistics were calculated in PAUP* 4.0 [42]. Phylogenies were estimated using maximum parsimony (MP), maximum likelihood (ML), and Bayesian methods.

### Phylogenetic analysis -- maximum parsimony

Parsimony analysis was conducted with TNT version 1.1 [43]. and summary statistics including CI (consistency index; [44]) and RI (retention index; [45]) were calculated using the Stats.run script available online at the TNT wiki (http://tnt.insectmuseum.org/index.php/Scripts). The tree search was conducted with the "mult" command, using 100 random addition runs as starting points, each followed by branch swapping via TBR (tree bisection and regrafting). After calculating summary statistics, the collection of most-parsimonious trees was summarized using combinable components (Bremer consensus tree), strict consensus (Nelsen consensus tree), and majority-rule consensus [43]. For each consensus tree, branch support was calculated using Bremer support [46,47], also known as decay index [48], and non-parametric bootstrapping [49]. Bremer support was calculating using the script KWBremer.run (provided by Kipling Will, personal communication), a modified version of Bremer.run available on the TNT scripts page. Bootstrapping was conducted with the "resample" command, using 100 bootstrap replicates.

### Phylogenetic analysis - maximum likelihood

Initial ML analysis was conducted using the online RAxML server [50] at http://phylobench.vital-it.ch/raxml-bb/ using defaults, and adding the option to estimate proportion of invariant sites.

More detailed ML analysis was conducted with Modeltest [51,52] and the PHYML [53] server online at http://mobyle.pasteur.fr/cgi-bin/portal.py?form=phyml. The standard Modeltest PAUP block was used to assess the likelihood of the sequence data as explained by a neighbor-joining (NJ) tree estimated from the data by PAUP and 56 different substitution models. The hierarchical likelihood ratio test (hLRT) selected HKY+I+G as the best model, and the Akaike Information Criterion (AIC) selected GTR+I+G. Three PHYML runs were conducted, the first using the specific parameters selected by Modeltest for HKY+I+G, the second using the specific parameters selected for GTR+I+G, and finally a run in which the GTR+I+G model was selected, but all parameters were estimated during the analysis. All runs were conducted with 100 bootstrap replicates, and majority-rule consensus trees with bootstrap branch support were calculated by PHYML.

### Phylogenetic analysis - Bayesian

MrModelTest [54] was used to assess the likelihood of the 24 sequence evolution models available in MrBayes. Again, hLRT selected HKY+I+G and AIC selected GTR+I+G; however, the point of Bayesian analysis is to sample trees (topologies and branch lengths) as well as substitution model parameters from the joint posterior distribution of trees and models, so no specific sequence evolution model was specified for MrBayes beyond the generic GTR+I+G with all parameters to be estimated during the run. MrBayes [55,56], available at http://mrbayes.csit.fsu.edu/, was used to conduct the phylogenetic analysis. Default parameters for estimation under a GTR+I+G model were used, with uniform priors set on the base frequencies and rate matrix, proportion of invariant sites, and topology. The prior on branch lengths was exponential with rate (alpha) = 10.0 and four categories were used to approximate gamma-distributed rate variation. Two independent runs were conducted of 1,000,000 generations each, with trees sampled every 1000 generations. The first 50% of each run was discarded as burnin, and the remaining 1000 saved trees were summarized using majority rule consensus. The standard deviation of split frequencies between the two runs stabilized at about 0.02, indicating that the runs had successfully converged and were sampling from the same posterior. There was some chance that estimating the full suite of parameters for a GTR+I+G model might be overly ambitious. Therefore, a second MrBayes analysis was performed using the same parameters, except with a maximally simple Jukes-Cantor (JC) model with no sequence evolution parameters estimated.

### Divergence Time Estimation

Inspection of the ML and Bayesian consensus trees indicated approximately clocklike behavior. Therefore tests were conducted to see if the hypothesis of a global molecular clock would be rejected by the data. The first set of tests was conducted in PAUP. The consensus tree from the GTR+I+G MrBayes run was manually rooted using the wild ass as outgroup. It was loaded into PAUP and its likelihood was measured for sequence evolution models constrained, and not constrained, to a global clock. The likelihoods were then compared to test for statistically significant difference using a likelihood ratio test with 62 degrees of freedom (number of taxa - 2). The test was repeated using 3 different models of sequence evolution: the HKY+I+G model selected by Modeltest, the GTR+I+G model selected by MrModeltest, and the posterior mean parameters of the GTR+I+G analysis selected by MrBayes.

The global clock hypothesis was also tested using the somewhat different procedures in the baseml program in PAML [57]. Here, the likelihood of the data with and without a global clock was estimated for the rooted Bayesian consensus tree using the GTR (termed "REV" in PAML) +I+G model where baseml estimates the optimal substitution model parameters for each analysis.

Following the decision that the assumption of a global clock was defensible, divergence times were estimated using r8s [58,59] and BEAST [26]. The primary goal of the analysis was to bracket the time of divergence of the horse breed mtDNA sequences; a completely thorough molecular dating exercise was not attempted here, as this would take a separate complex study at least involving the incorporation of many partial mtDNA sequences available from subfossil equines [60]. Therefore, the divergence time of the horse/ass clade as estimated from the fossil record was used as the only constraint. Since bracketing the divergence time was the major goal, the maximum (3.5 mybp) and minimum (1.0 mybp) possible divergence times based on the fossil record [8] were used as the constraints. For a maximum-divergence-time r8s run the horse/ass split was fixed at 3.5 mybp, and for the minimum-divergence-time r8s run, it was fixed to 1.0 mybp. R8s was run using the default Langley-Fitch (molecular clock) method of estimating divergence times.

Divergence time estimation was also conducted using a strict global clock assumption in BEAST, in order to get a more reasonable sense of the variability in divergence times for horse lineages. It is admitted that the choice of prior used in this analysis is fairly subjective and thus the results are heuristic rather than firm conclusions. Utilizing the reasonable assumption that the true divergence time of horse and ass is more likely to be in the middle of the 3.5-1.0 mybp range than at the edges, the prior on the divergence time of horse and ass was set to be normally distributed with a mean of 2.25 mybp, and with the standard deviation set to 0.3125 my, so that "maximum" and "minimum" divergence times occurred 4 standard deviations above and below the mean. All other parameters were allowed to vary during BEAST's sampling routine, using default priors, except as follows: the substitution model was HKY+I+G with 4 gamma rate categories, estimated base frequencies, and uniform prior of the substitution rate sampling between 0 to 1. The convergence of the MCMC analysis was judged to be adequate after inspection of the run in Tracer. The first 10% of the BEAST MCMC run was discarded as burn-in, and the remaining samples were summarized using TreeAnnotator. The resulting ultrametric trees were displayed in FigTree (all programs available with BEAST at: http://beast.bio.ed.ac.uk/Main_Page).

The Bayesian skyline plot method implemented in BEAST was used to estimate past population dynamics through time from the 63 whole mtDNA horse sequences. A piecewise linear model and the HKY+I+G substitution model was chosen and the substitution rate (estimated in the divergence time analysis) was set by a normally distributed prior with a mean of 0.074 subst/pos/Mya and a standard deviation of 0.01 subst/pos/Mya. Each MCMC run was conducted on 10 million iterations and the first 10% of each run was discarded as burn-in. The results of three independent runs were verified in Tracer and combined with Treeannotator. BSP were drawn with Tracer using linear change mode and the combined tree file. The effective population size was estimated assuming a generation time of 10 years [61]. The same parameters were used to run the MCMC for a constant size coalescence model. In order to compare the BSP and constant size model and to see if one model is favoured over the other the Bayes factor (BF) was calculated using the BF analysis option implemented in Tracer.

## Authors' contributions

SL and MH conceived and designed the experiments. SL performed the experiments. NJM and SL analysed the data. MR provided DNA samples. All authors contributed to writing the final version of the paper. All authors read and approved the final manuscript.

## Supplementary Material

Additional file 1 **Figure S1**. Majority-rule consensus tree generated using all 66 full horse mtDNA sequences. **Table S1**. Sample information for the 60 whole mtDNA genomes sequenced in this study. **Table S2**. Summary statistics of the BWA mapping and consensus calling. **Table S3**. Genbank record IDs and full names from the NCBI database are given for 7 previously published sequences taken from Genbank. **Table S4**. Summary statistics for the different phylogenetic analyses. (A) parsimony analyses (B) ML and Bayesian analyses. **Table S5**. Tests of the global molecular clock with likelihood ratio (LR) tests. Click here for file
