# Supplementary material for: Whole mitochondrial genome sequencing of domestic horses reveals incorporation of extensive wild horse diversity during domestication
Source: BMC Evol Biol. 2011 Nov 14;11:328. doi: 10.1186/1471-2148-11-328 (PMC3247663; doi:10.1186/1471-2148-11-328)
Supplement: Additional file 1 — Figure S1. Majority-rule consensus tree generated using all 66 full horse mtDNA sequences. Table S1. Sample information for the 60 whole mtDNA genomes sequenced in this study. Table S2. Summary statistics of the BWA mapping and consensus calling. Table S3. Genbank record IDs and full names from the NCBI database are given for 7 previously published sequences taken from Genbank. Table S4. Summary statistics for the different phylogenetic analyses. (A) parsimony analyses (B) ML and Bayesian analyses. Table S5. Tests of the global molecular clock with likelihood ratio (LR) tests. [file 1471-2148-11-328-S1.PDF]

Supplements

Whole mitochondrial genome sequencing of domestic horses reveals incorporation of extensive wild horse diversity during domestication

Sebastian Lippold[1§], Nicholas J. Matzke[2], Monika Reissmann[3] and Michael Hofreiter[4]

[1] Department of Evolutionary Genetics, Max Planck Institute for Evolutionary Anthropology, Deutscher Platz 6, 04103 Leipzig, Germany

[2] Center for Theoretical Evolutionary Genomics, Department of Integrative Biology, University of California, Berkeley, 4151 Valley Life Sciences Building, Berkeley, CA, USA

[3] Department for Crop and Animal Sciences, Humboldt University Berlin, Invalidenstr. 42, 10115 Berlin, Germany

[4] Department of Biology, University of York, Wentworth Way, Heslington, York YO10 5DD, UK

[§]Corresponding author

Email addresses:

SL: sebastian_lippold@eva.mpg.de
NJM: matzke@berkeley.edu
MR: monika.reissmann@rz.hu-berlin.de
MH: michi@palaeo.eu

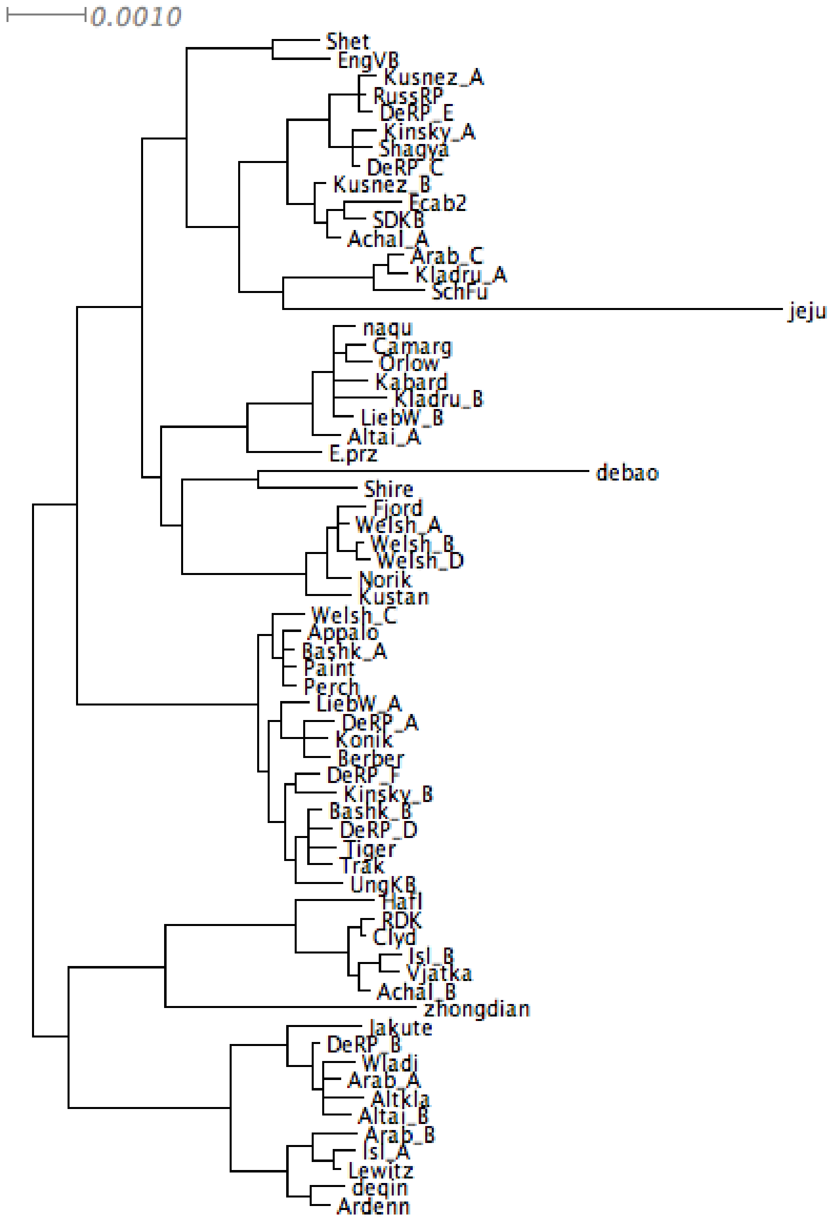

Figure S1: Majority-rule consensus tree generated using all 66 full horse mtDNA sequences. The tree was generated with MrBayes 3.1.2, using a GTR+I+G model of sequence evolution, with all parameters estimated during the run, using default priors. The analysis consisted of two independent runs of one million generations each, with trees sampled every 1000 steps; the standard deviation of split frequencies between the runs at the end of the analysis was 0.009271. The last 500 trees of each run were summarized to produce the consensus tree. MrBayes runs with other parameters (e.g. Jukes-Cantor, HKY) produced similar consensus trees, as did ML algorithms. Three sequences, “jeju”, “debao”, and “zhongdian”, are anomalous and were excluded from futher analysis (for further discussion see main text).

Table S1: Sample information for the 60 whole mtDNA genomes sequenced in this study.

ID	Breed	Abbreviation	General region	GenBank accession number
0902	Akhal-Teke	Achal_A	Central Asia	HQ439441
1352	Akhal-Teke	Achal_B	Central Asia	HQ439442
1416	Altai	Altai_A	Central Asia	HQ439443
2310	Altai	Altai_B	Central Asia	HQ439444
0851	Kladruber	Altkla	Central Europe	HQ439445
0453	Appaloosa	Appalo	North America	HQ439446
0910	Arab	Arab_A	Middle East	HQ439447
3020	Arab	Arab_B	Middle East	HQ439448
3023	Arab	Arab_C	Middle East	HQ439449
0698	Ardennais	Ardenn	West Europe	HQ439450
2941	Bashkir Curly	Bashk_A	North America	HQ439451
2942	Bashkir Curly	Bashk_B	North America	HQ439452
1538	Barb	Berber	North Africa	HQ439453
0214	Camargue	Camarg	West Europe	HQ439454
2950	Clydesdale	Clyd	West Europe	HQ439455
1531	German Sport Horse	DeRP_A	Central Europe	HQ439456
1286	Hanoverian	DeRP_B	Central Europe	HQ439457
0058	Holstein	DeRP_C	Central Europe	HQ439458
1287	Oldenburg	DeRP_D	Central Europe	HQ439459
0006	Westphalian	DeRP_E	Central Europe	HQ439460
1289	German Riding Pony	DeRP_F	Central Europe	HQ439461
0055	English Thoroughbred	EngVB	West Europe	HQ439462
2881	Norwegian Fjord	Fjord	North Europe	HQ439463
2753	Haflinger	Hafl	Central Europe	HQ439464
2240	Icelandic horse	Isl_A	North Europe	HQ439465
2898	Icelandic horse	Isl_B	North Europe	HQ439466
0493	Yakut	Jakute	East Asia	HQ439467
3006	Kabardin	Kabard	East Europe	HQ439468
0771	Kinsky horse	Kinsky_A	Central Europe	HQ439469
2686	Kinsky horse	Kinsky_B	Central Europe	HQ439470
0827	Kladruber	Kladru_A	Central Europe	HQ439471
0837	Kladruber	Kladru_B	Central Europe	HQ439472
2972	Konik	Konik	Central Europe	HQ439473
2275	Kuznet	Kusnez_A	East Europe	HQ439474
2280	Kuznet	Kusnez_B	East Europe	HQ439475
0391	Kustanai	Kustan	Central Asia	HQ439476
2643	Lewitzer	Lewitz	Central Europe	HQ439477
0582	Liebenthaler	LiebW_A	Central Europe	HQ439478
0624	Liebenthaler	LiebW_B	Central Europe	HQ439479
0263	Noriker	Norik	Central Europe	HQ439480
2266	Orlov Trotter	Orlow	East Europe	HQ439481

ID	Breed	Abbreviation	General region	GenBank accession number
0505	Painted Horse	Paint	North America	HQ439482
0901	Percheron	Perch	West Europe	HQ439483
0078	Przewalski's horse	E.prz	East Asia	HQ439484
0908	Russian Riding Horse	RussRP	East Europe	HQ439486
D34	Black Forest	SchFu	Central Europe	HQ439487
0293	Shagya Arab	Shagya	Central Europe	HQ439488
2747	Shetland	Shet	West Europe	HQ439489
0251	Shire	Shire	West Europe	HQ439490
0357	Rhineland Heavy Draft	SDKB	Central Europe	HQ439491
2657	Spotted horse	Tiger	Central Europe	HQ439492
1019	Trakehner	Trak	Central Europe	HQ439493
0209	Hungarian Coldblood	UngKB	Central Europe	HQ439494
2265	Viatka	Vjatka	East Europe	HQ439495
0407	Welsh Cob (Section D)	Welsh_A	West Europe	HQ439496
2966	Welsh Pony (Section A)	Welsh_B	West Europe	HQ439497
0419	Welsh Pony (Section B)	Welsh_C	West Europe	HQ439498
2944	Welsh Pony (Section B)	Welsh_D	West Europe	HQ439499
2261	Vladimir Heavy Draught	Wladi	East Europe	HQ439500

Table S2: Summary statistics of the BWA mapping and consensus calling. N = positions that do not fulfill the final consensus call criteria (see methods). DR = duplicate removal

ID	Breed	# mt reads		average coverage	# N / mt genome [%]
		before DR	after DR		
0902	Akhal-Teke	18,594	13,122	60.1	0.00%
1352	Akhal-Teke	13,990	9,763	44.7	0.00%
1416	Altai	22,222	14,958	68.5	0.00%
2310	Altai	18,335	12,115	55.5	0.00%
0851	Kladruber	17,442	11,786	54.0	0.00%
0453	Appaloosa	23,689	15,945	73.0	0.00%
0910	Arab	21,223	13,924	63.7	0.00%
3020	Arab	13,085	9,292	42.5	0.01%
3023	Arab	23,160	14,511	66.4	0.00%
0698	Ardennais	19,467	13,273	60.8	0.01%
2941	Bashkir Curly	24,145	14,751	67.5	0.00%
2942	Bashkir Curly	15,882	10,714	49.1	0.00%
1538	Barb	14,156	10,079	46.1	0.02%
0214	Camargue	21,520	15,042	68.9	0.00%
2950	Clydesdale	27,966	17,858	81.8	0.00%
1531	German Sport Horse	11,537.	8,479	38.8	0.14%
1286	Hanoverian	9,714	7,151	32.7	0.57%
0058	Holstein	12,412	9,247	42.3	0.11%
1287	Oldenburg	11,601	8,544	39.1	0.24%
0006	Westphalian	12,098	8,951	41.0	0.57%
1289	German Riding Pony	15,230	10,785	49.4	0.01%
0055	English Thoroughbred	11,225	8,351	38.2	0.75%
2881	Norwegian Fjord	21,957	15,243	69.8	0.00%
2753	Haflinger	14,918	10,261	47.0	0.00%
2240	Icelandic horse	17,294	11,953	54.7	0.00%
2898	Icelandic horse	16,502	11,529	52.8	0.00%
0493	Yakut	24,074	16,059	73.5	0.00%
3006	Kabardin	19,126	13,108	60.0	0.00%
0771	Kinsky horse	24,864	16,409	75.1	0.00%
2686	Kinsky horse	19,449	12,338	56.5	0.00%
0827	Kladruber	20,102	12,574	57.6	0.00%
0837	Kladruber	28,982	16,518	75.6	0.00%
2972	Konik	10,173	7,734	35.4	0.05%
2275	Kuznet	12,701	8,849	40.5	0.00%
2280	Kuznet	20,305	12,741	58.3	0.00%
0391	Kustanai	30,369	17,181	78.7	0.00%
2643	Lewitzer	18,778	12,381	56.7	0.00%
0582	Liebenthaler	12,411	8,423	38.6	0.00%
0624	Liebenthaler	17,608	12,112	55.5	0.09%

ID	Breed	# mt reads		average coverage	# N / mt genome [%]
		before DR	after DR		
0263	Noriker	11,080	7,291	33.4	0.00%
2266	Orlov Trotter	11,031	7,206	33.0	0.01%
0901	Percheron	22,942	14,654	67.1	0.00%
0078	Przewalskii's horse	7,183	5,564	25.5	0.01%
1282	Rhineland Heavy Draft	13,468	9,874	45.2	0.32%
0908	Russian Riding Horse	23,056	14,297	65.5	0.00%
D34	Black Forest	12,993	8,573	39.2	0.00%
0293	Shagya Arab	17,628	12,409	56.8	0.02%
2747	Shetland	28,210	15,791	72.3	0.00%
0251	Shire	16,732	11,924	54.6	0.19%
0357	Rhineland Heavy Draft	18,887	12,597	57.7	0.00%
2657	Spotted horse	17,894	11,728	53.7	0.00%
1019	Trakehner	12,438	9,431	43.2	0.01%
0209	Hungarian Coldblood	10,991	7,160	32.8	0.01%
2265	Viatka	5,666	3,079	14.1	0.01%
0407	Welsh Cob (Section D)	22,607	15,223	69.7	0.00%
2966	Welsh Pony (Section A)	26,377	15,841	72.5	0.00%
0419	Welsh Pony (Section B)	16,636	11,398	52.2	0.00%
2944	Welsh Pony (Section B)	18,340	12,309	56.4	0.00%
2261	Vladimir Heavy Draught	17,367	12,175	55.7	0.01%

Table S3: Genbank record IDs and full names from the NCBI database are given for 7 previously published sequences taken from Genbank. Sequences marked with * exhibited unusually long branches in the phylogenetic analysis and were excluded from analysis in this study.

Species	Breed	GenBank	Reference
E. asinus (outgroup)		X97337.1	[1]
E. caballus	unknown, from Sweden	X79547.1	[2]
E. caballus	Deqin	EF597514.1	[3]
E. caballus	Naqu	EF597513.1	[3]
E. caballus	Zhongdian	EF597512.1*	[3]
E. caballus	Debao	EU939445.3*	[4]
E. caballus	Cheju	AY584828.1*	not published

Table S4: Summary statistics for the different phylogenetic analyses. (A) For parsimony analyses, the number of steps for the most parsimonious trees are reported, as are the CI, RI, and RCI (CI * RI). (B) For ML and Bayesian analyses, the log-likelihood values are reported for either the best tree (ML) or the arithmetic mean of the sampled posterior trees (Bayesian). The tree length is given where reported. The model substitution parameters are either the estimated parameters (arithmetic means from the posterior distribution for the GTR Bayesian analysis) or the user-specified parameters (for the PHYML analysis using Modeltest-derived substitution parameters and for the Bayesian Jukes-Cantor analysis).

**A. Parsimony Results:**						
**Description**	**Branch supports calculated**	**Summary statistics**				
		**# optimal trees**	**# steps**	**CI**	**RI**	**RCI**
TNT, mult 100, SPR, hold 400; 276,846,227 rearrangments tried	Bremer and 100 bootstraps on Nelson, strict, and majority rule consensus	353	707	0.52	0.88	0.46
TNT, aquickie script; 116,931,722 rearrangments tried	100 bootstraps on Nelson consensus	n/a	707	0.50	0.87	0.44

B. Likelihood/Bayesian Results:

Description	Branch supports calculated	optimal trees	lnL	AC	AG	AT	CG	CT	GT	freqA	freqC	freqG	freqT	alpha	prop invr	tree length
RAxML blackbox, defaults + estimate proportion of invariant sites	100 bootstraps on best ML tree	1	-31757.0	0.42	45.66	0.73	0.92	29.95	1	0.32	0.28	0.13	0.26	0.87	0.79	1.32
PHYML, HKY+I+G model from Modeltest	100 bootstraps on best ML tree	1	-31797.7	1	21.62	1	1	21.62	1	0.32	0.28	0.13	0.26	0.85	0.78	n/a
PHYML, HKY+I+G model, estimate parameters	100 bootstraps on best ML tree	1	-31765.9	1	45	1	1	45	1	0.32	0.28	0.13	0.26	0.82	0.78	n/a
PHYML, GTR+I+G model, estimate parameters	100 bootstraps on best ML tree	1	-31753	0.42	46.61	0.82	0.98	30.74	1	0.32	0.28	0.13	0.26	0.81	0.79	n/a
MrBayes, GTR+I+G, estimate parameters	1000 posterior samples	1000	-32893.7	0.01	0.57	0.01	0.02	0.38	0.01	0.32	0.28	0.14	0.26	0.08	0.78	9.41
MrBayes, Jukes-Cantor	1000 posterior samples	1000	-34454.6	1	1	1	1	1	1	0.25	0.25	0.25	0.25	n/a	n/a	0.12

Table S5: Tests of the global molecular clock with likelihood ratio (LR) tests.

Substitution model	Program	-lnL, no clock	-lnL, clock	difference	Chi-sq	degrees of freedom	p-value
HKY+I+G, from Modeltest	PAUP	32437.4	32480.8	43.4	86.7	62	0.021
GTR+I+G, from MrModeltest	PAUP	31787.4	31828.2	40.8	81.6	62	0.049
GTR+I+G, from MrBayes	PAUP	33014.8	33057.5	42.7	85.4	62	0.026
GTR+I+G, estimated on branches	baseML	31878.6	31918.8	40.3	80.5	62	0.057

References

1. Xu XF, Gullberg A, Arnason U: **The complete mitochondrial DNA (mtDNA) of the donkey and mtDNA comparisons among four closely related mammalian species-pairs**. *Journal of Molecular Evolution* 1996, **43**(5):438-446.
2. Xu XF, Arnason U: **The Complete Mitochondrial-DNA Sequence of the Horse, Equus-Caballus - Extensive Heteroplasmy of the Control Region**. *Gene* 1994, **148**(2):357-362.
3. Xu S, Luosang J, Hua S, He J, Ciren A, Wang W, Tong X: **High altitude adaptation and phylogenetic analysis of Tibetan horse based on the mitochondrial genome**. *Journal of Genetics and Genomics* 2007, **34**(8):720-729.
4. Jiang QY, Wei YM, Huang YN, Jiang HS, Guo YF, Lan GQ, Liao DJ: **The complete mitochondrial genome and phylogenetic analysis of the Debao pony (*Equus caballus*)**. *Molecular Biology Reports* 2011, **38**(1):593-599.
